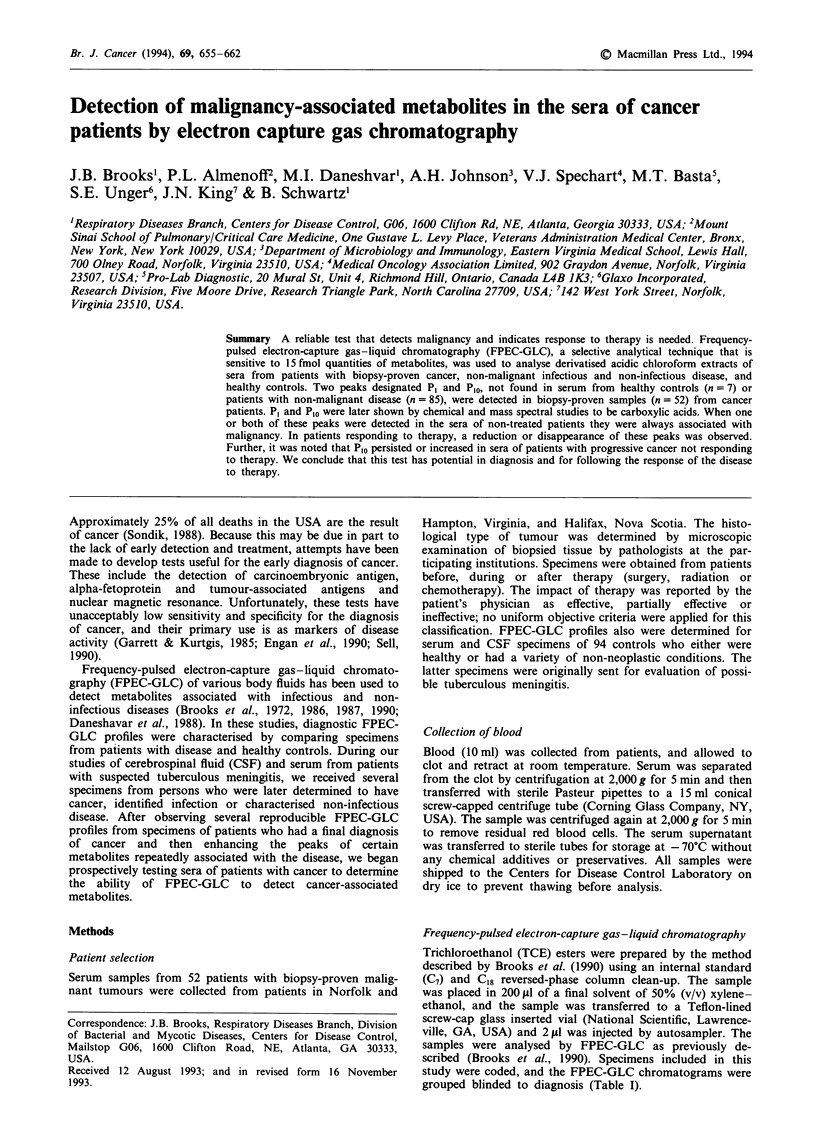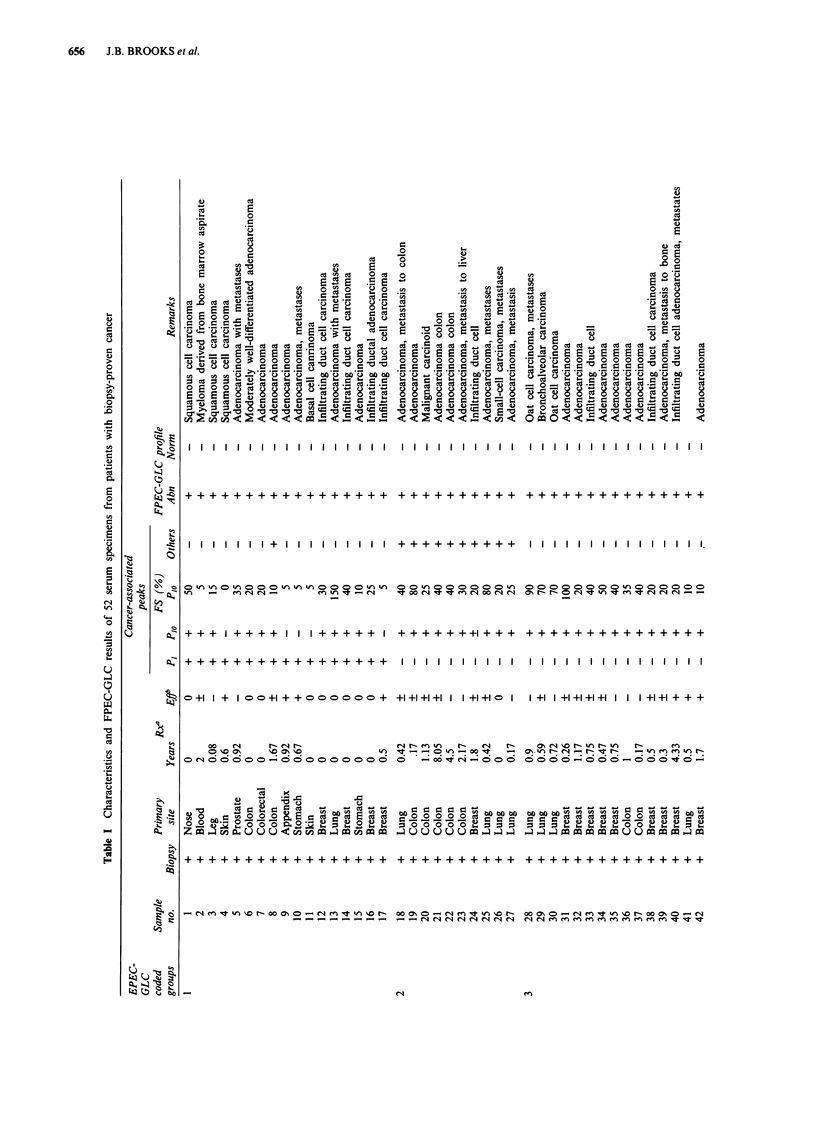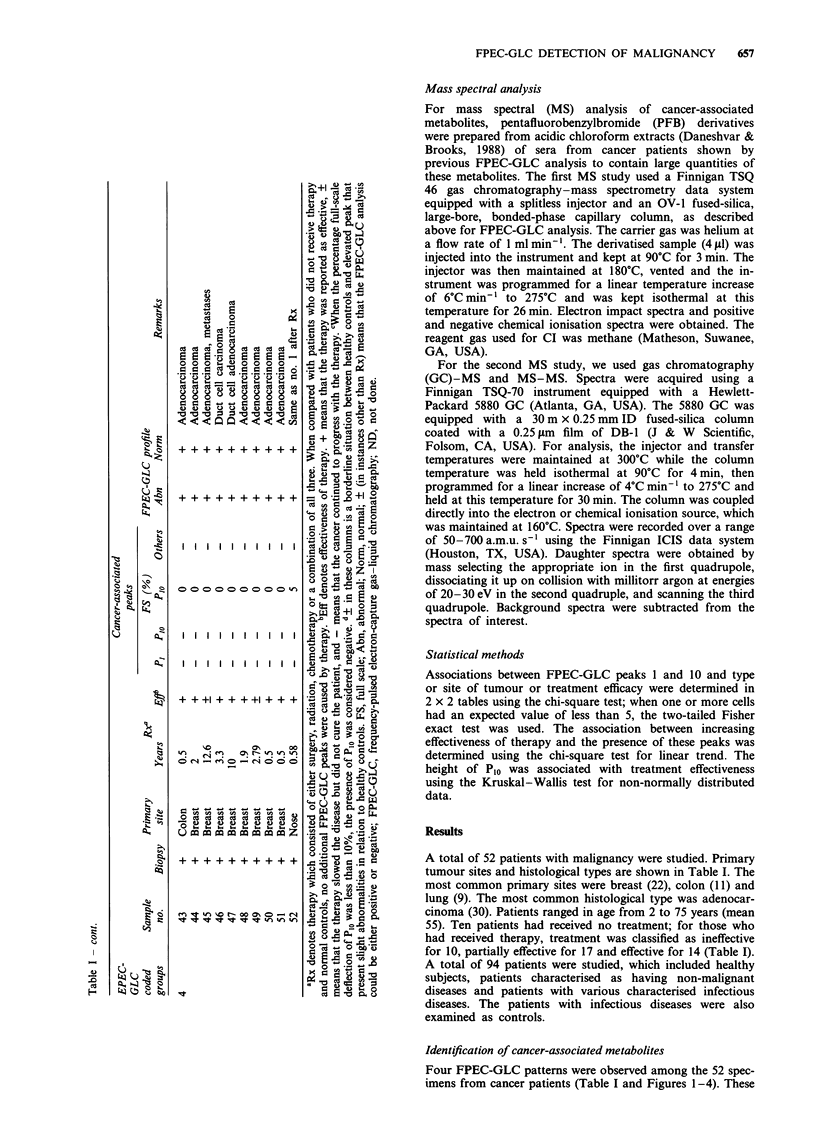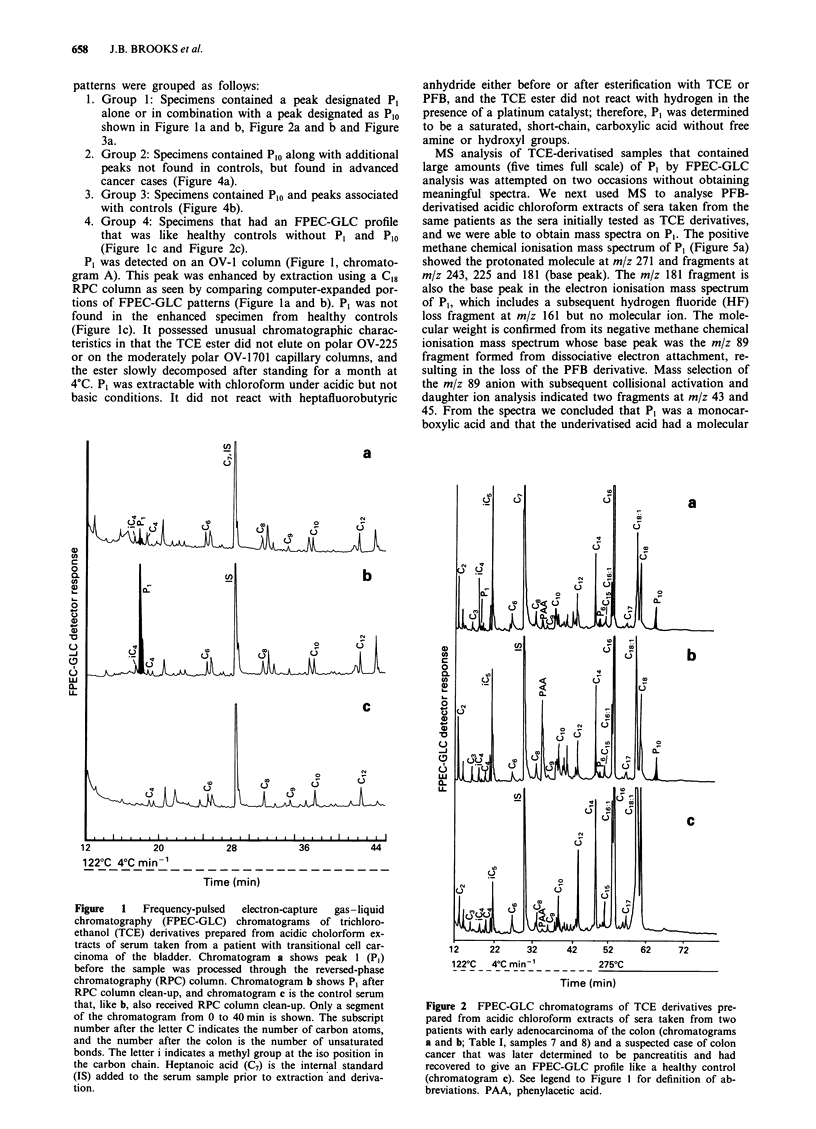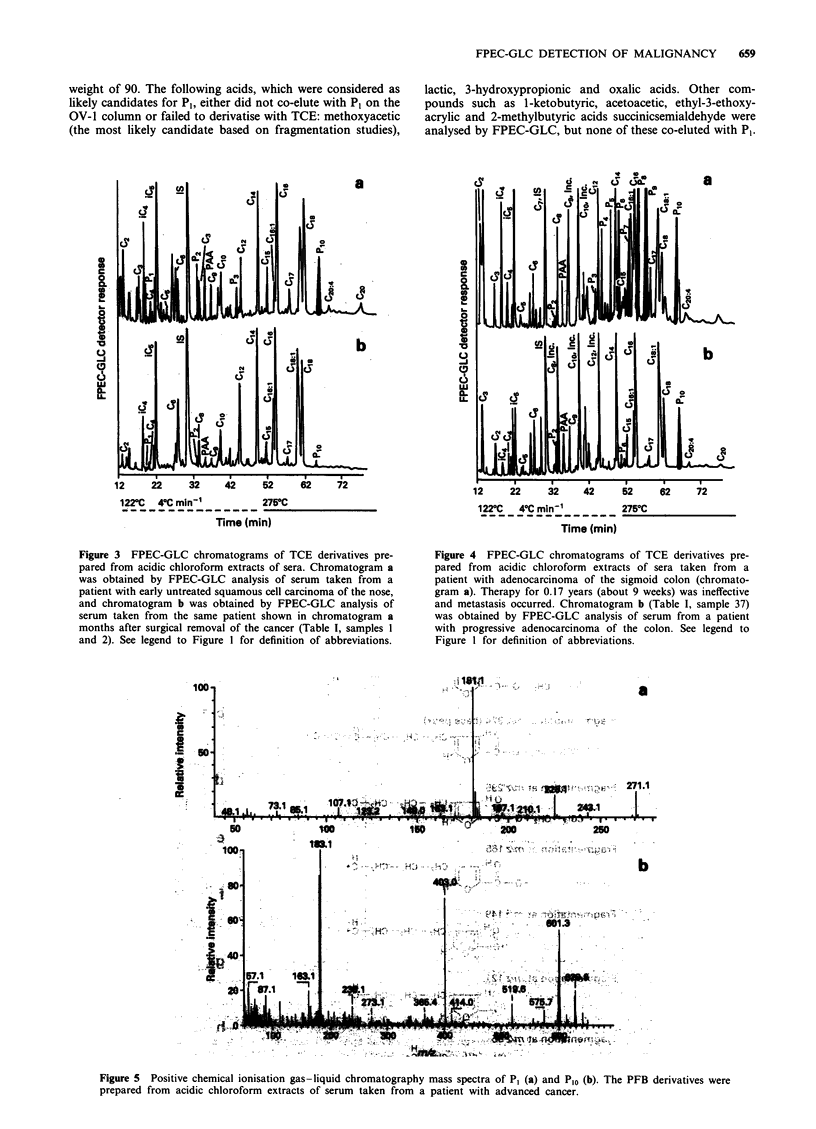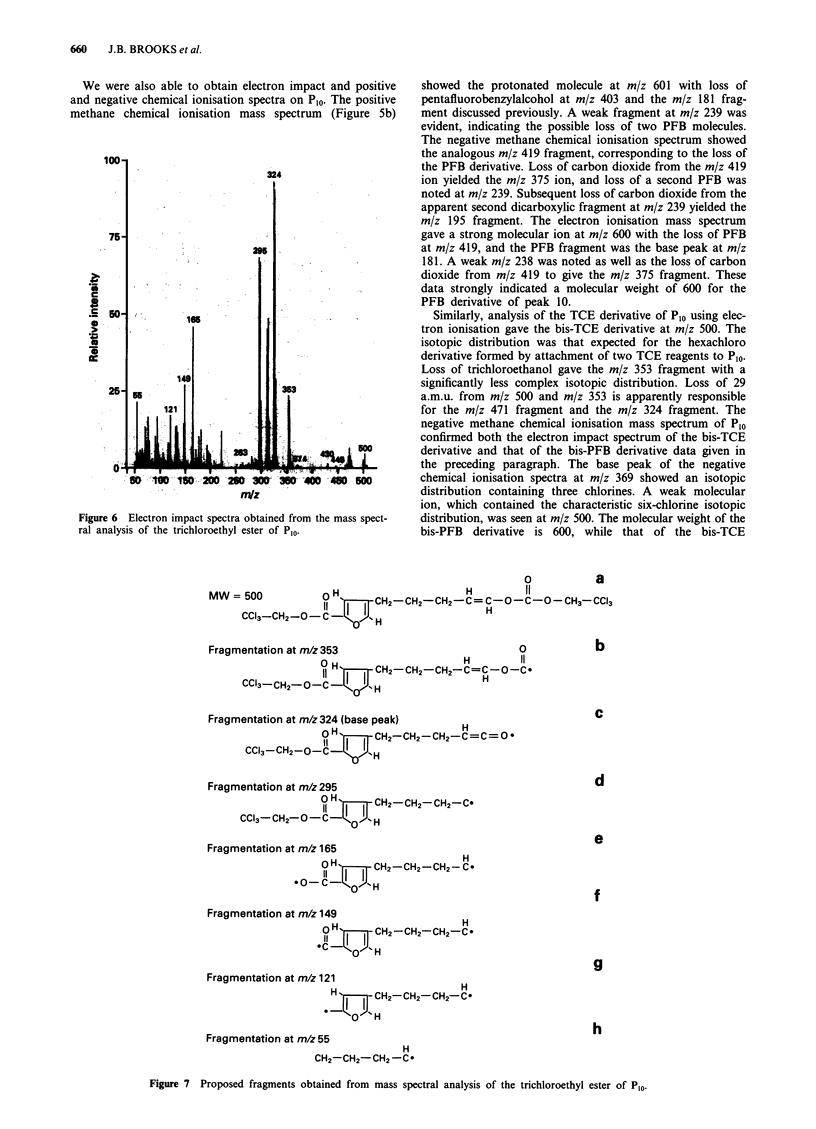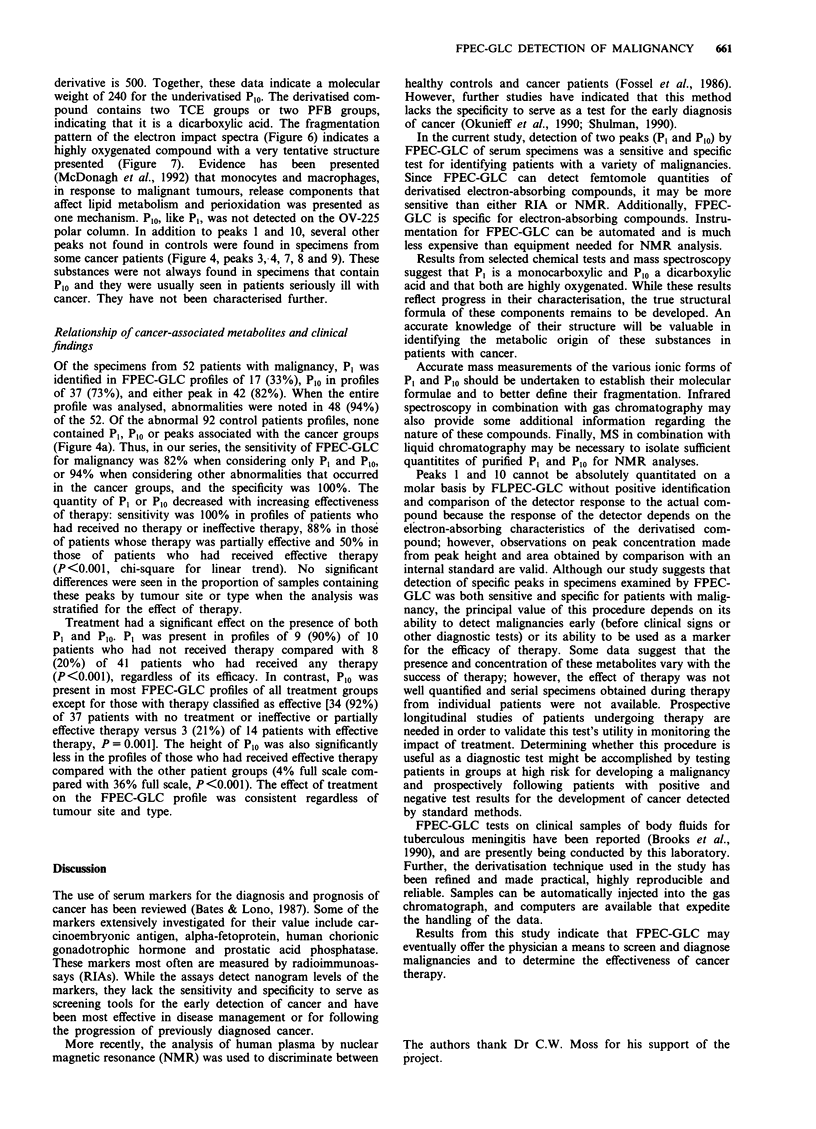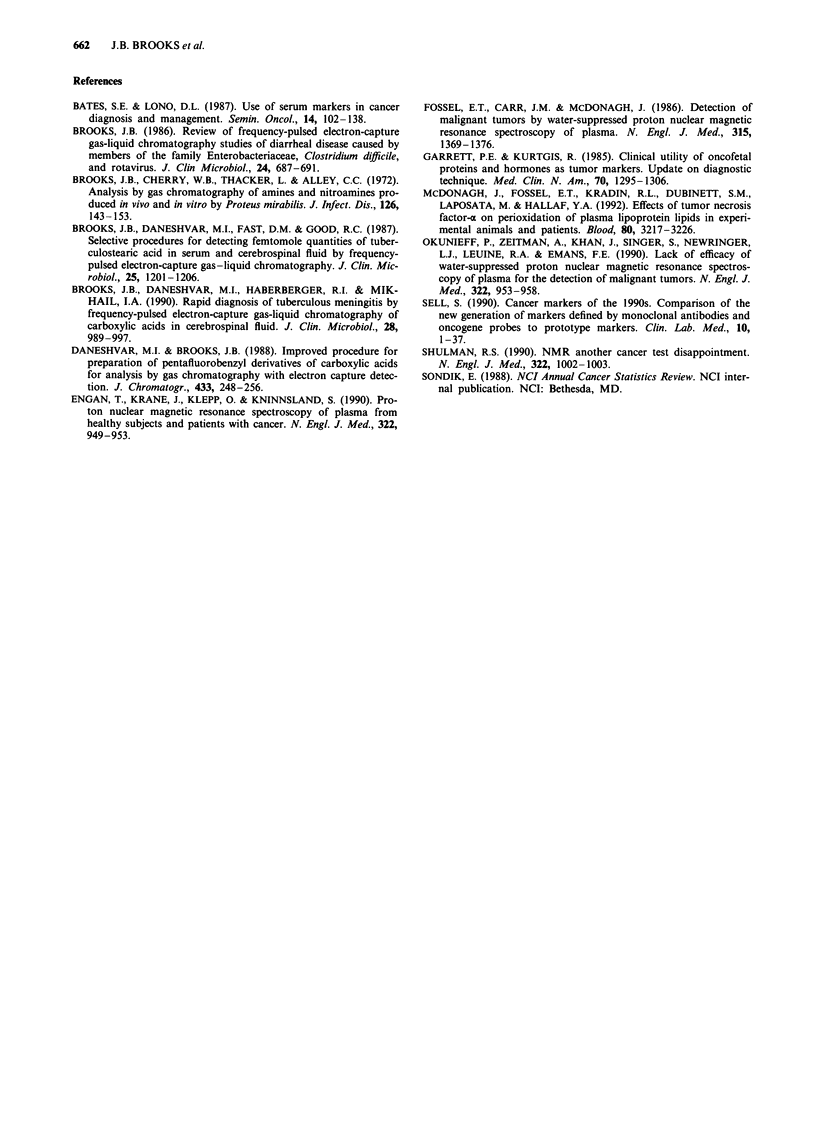# Detection of malignancy-associated metabolites in the sera of cancer patients by electron capture gas chromatography.

**DOI:** 10.1038/bjc.1994.127

**Published:** 1994-04

**Authors:** J. B. Brooks, P. L. Almenoff, M. I. Daneshvar, A. H. Johnson, V. J. Spechart, M. T. Basta, S. E. Unger, J. N. King, B. Schwartz

**Affiliations:** Respiratory Diseases Branch, Centers for Disease Control, Atlanta, Georgia 30333.

## Abstract

A reliable test that detects malignancy and indicates response to therapy is needed. Frequency-pulsed electron-capture gas-liquid chromatography (FPEC-GLC), a selective analytical technique that is sensitive to 15 fmol quantities of metabolites, was used to analyse derivatised acidic chloroform extracts of sera from patients with biopsy-proven cancer, non-malignant infectious and non-infectious disease, and healthy controls. Two peaks designated P1 and P10, not found in serum from healthy controls (n = 7) or patients with non-malignant disease (n = 85), were detected in biopsy-proven samples (n = 52) from cancer patients. P1 and P10 were later shown by chemical and mass spectral studies to be carboxylic acids. When one or both of these peaks were detected in the sera of non-treated patients they were always associated with malignancy. In patients responding to therapy, a reduction or disappearance of these peaks was observed. Further, it was noted that P10 persisted or increased in sera of patients with progressive cancer not responding to therapy. We conclude that this test has potential in diagnosis and for following the response of the disease to therapy.


Br. J. Cancer (1994), 69, 655–662 © Macmillan Press Ltd., 1994

### Detection of malignancy-associated metabolites in the sera of cancer patients by electron capture gas chromatography

J.B. Brooks[1], P.L. Almenoff[2], M.I. Daneshvar[1], A.H. Johnson[3], V.J. Spechart[4], M.T. Basta[5], S.E. Unger[6], J.N. King[7] & B. Schwartz[1]

[1]*Respiratory Diseases Branch, Centers for Disease Control, G06, 1600 Clifton Rd, NE, Atlanta, Georgia 30333, USA;* [2]*Mount Sinai School of Pulmonary/Critical Care Medicine, One Gustave L. Levy Place, Veterans Administration Medical Center, Bronx, New York, New York 10029, USA;* [3]*Department of Microbiology and Immunology, Eastern Virginia Medical School, Lewis Hall, 700 Olney Road, Norfolk, Virginia 23510, USA;* [4]*Medical Oncology Association Limited, 902 Graydon Avenue, Norfolk, Virginia 23507, USA;* [5]*Pro-Lab Diagnostic, 20 Mural St, Unit 4, Richmond Hill, Ontario, Canada L4B 1K3;* [6]*Glaxo Incorporated, Research Division, Five Moore Drive, Research Triangle Park, North Carolina 27709, USA;* [7]*142 West York Street, Norfolk, Virginia 23510, USA.*

**Summary** A reliable test that detects malignancy and indicates response to therapy is needed. Frequency-pulsed electron-capture gas–liquid chromatography (FPEC-GLC), a selective analytical technique that is sensitive to 15 fmol quantities of metabolites, was used to analyse derivatised acidic chloroform extracts of sera from patients with biopsy-proven cancer, non-malignant infectious and non-infectious disease, and healthy controls. Two peaks designated $P_1$ and $P_{10}$, not found in serum from healthy controls ($n = 7$) or patients with non-malignant disease ($n = 85$), were detected in biopsy-proven samples ($n = 52$) from cancer patients. $P_1$ and $P_{10}$ were later shown by chemical and mass spectral studies to be carboxylic acids. When one or both of these peaks were detected in the sera of non-treated patients they were always associated with malignancy. In patients responding to therapy, a reduction or disappearance of these peaks was observed. Further, it was noted that $P_{10}$ persisted or increased in sera of patients with progressive cancer not responding to therapy. We conclude that this test has potential in diagnosis and for following the response of the disease to therapy.

Approximately 25% of all deaths in the USA are the result of cancer (Sondik, 1988). Because this may be due in part to the lack of early detection and treatment, attempts have been made to develop tests useful for the early diagnosis of cancer. These include the detection of carcinoembryonic antigen, alpha-fetoprotein and tumour-associated antigens and nuclear magnetic resonance. Unfortunately, these tests have unacceptably low sensitivity and specificity for the diagnosis of cancer, and their primary use is as markers of disease activity (Garrett & Kurtgis, 1985; Engan *et al.*, 1990; Sell, 1990).

Frequency-pulsed electron-capture gas–liquid chromatography (FPEC-GLC) of various body fluids has been used to detect metabolites associated with infectious and non-infectious diseases (Brooks *et al.*, 1972, 1986, 1987, 1990; Daneshavar *et al.*, 1988). In these studies, diagnostic FPEC-GLC profiles were characterised by comparing specimens from patients with disease and healthy controls. During our studies of cerebrospinal fluid (CSF) and serum from patients with suspected tuberculous meningitis, we received several specimens from persons who were later determined to have cancer, identified infection or characterised non-infectious disease. After observing several reproducible FPEC-GLC profiles from specimens of patients who had a final diagnosis of cancer and then enhancing the peaks of certain metabolites repeatedly associated with the disease, we began prospectively testing sera of patients with cancer to determine the ability of FPEC-GLC to detect cancer-associated metabolites.

### Methods

### *Patient selection*

Serum samples from 52 patients with biopsy-proven malignant tumours were collected from patients in Norfolk and Hampton, Virginia, and Halifax, Nova Scotia. The histological type of tumour was determined by microscopic examination of biopsied tissue by pathologists at the participating institutions. Specimens were obtained from patients before, during or after therapy (surgery, radiation or chemotherapy). The impact of therapy was reported by the patient's physician as effective, partially effective or ineffective; no uniform objective criteria were applied for this classification. FPEC-GLC profiles also were determined for serum and CSF specimens of 94 controls who either were healthy or had a variety of non-neoplastic conditions. The latter specimens were originally sent for evaluation of possible tuberculous meningitis.

### *Collection of blood*

Blood (10 ml) was collected from patients, and allowed to clot and retract at room temperature. Serum was separated from the clot by centrifugation at 2,000 *g* for 5 min and then transferred with sterile Pasteur pipettes to a 15 ml conical screw-capped centrifuge tube (Corning Glass Company, NY, USA). The sample was centrifuged again at 2,000 *g* for 5 min to remove residual red blood cells. The serum supernatant was transferred to sterile tubes for storage at −70°C without any chemical additives or preservatives. All samples were shipped to the Centers for Disease Control Laboratory on dry ice to prevent thawing before analysis.

### *Frequency-pulsed electron-capture gas–liquid chromatography*

Trichloroethanol (TCE) esters were prepared by the method described by Brooks *et al.* (1990) using an internal standard ($C_7$) and $C_{18}$ reversed-phase column clean-up. The sample was placed in 200 µl of a final solvent of 50% (v/v) xylene–ethanol, and the sample was transferred to a Teflon-lined screw-cap glass inserted vial (National Scientific, Lawrenceville, GA, USA) and 2 µl was injected by autosampler. The samples were analysed by FPEC-GLC as previously described (Brooks *et al.*, 1990). Specimens included in this study were coded, and the FPEC-GLC chromatograms were grouped blinded to diagnosis (Table I).

Correspondence: J.B. Brooks, Respiratory Diseases Branch, Division of Bacterial and Mycotic Diseases, Centers for Disease Control, Mailstop G06, 1600 Clifton Road, NE, Atlanta, GA 30333, USA.
Received 12 August 1993; and in revised form 16 November 1993.

*Mass spectral analysis*

For mass spectral (MS) analysis of cancer-associated metabolites, pentafluorobenzylbromide (PFB) derivatives were prepared from acidic chloroform extracts (Daneshvar & Brooks, 1988) of sera from cancer patients shown by previous FPEC-GLC analysis to contain large quantities of these metabolites. The first MS study used a Finnigan TSQ 46 gas chromatography–mass spectrometry data system equipped with a splitless injector and an OV-1 fused-silica, large-bore, bonded-phase capillary column, as described above for FPEC-GLC analysis. The carrier gas was helium at a flow rate of 1 ml $min^{-1}$. The derivatised sample (4 µl) was injected into the instrument and kept at 90°C for 3 min. The injector was then maintained at 180°C, vented and the instrument was programmed for a linear temperature increase of 6°C $min^{-1}$ to 275°C and was kept isothermal at this temperature for 26 min. Electron impact spectra and positive and negative chemical ionisation spectra were obtained. The reagent gas used for CI was methane (Matheson, Suwanee, GA, USA).

For the second MS study, we used gas chromatography (GC)–MS and MS–MS. Spectra were acquired using a Finnigan TSQ-70 instrument equipped with a Hewlett-Packard 5880 GC (Atlanta, GA, USA). The 5880 GC was equipped with a 30 m × 0.25 mm ID fused-silica column coated with a 0.25 µm film of DB-1 (J & W Scientific, Folsom, CA, USA). For analysis, the injector and transfer temperatures were maintained at 300°C while the column temperature was held isothermal at 90°C for 4 min, then programmed for a linear increase of 4°C $min^{-1}$ to 275°C and held at this temperature for 30 min. The column was coupled directly into the electron or chemical ionisation source, which was maintained at 160°C. Spectra were recorded over a range of 50–700 a.m.u. $s^{-1}$ using the Finnigan ICIS data system (Houston, TX, USA). Daughter spectra were obtained by mass selecting the appropriate ion in the first quadrupole, dissociating it up on collision with millitorr argon at energies of 20–30 eV in the second quadruple, and scanning the third quadrupole. Background spectra were subtracted from the spectra of interest.

*Statistical methods*

Associations between FPEC-GLC peaks 1 and 10 and type or site of tumour or treatment efficacy were determined in 2 × 2 tables using the chi-square test; when one or more cells had an expected value of less than 5, the two-tailed Fisher exact test was used. The association between increasing effectiveness of therapy and the presence of these peaks was determined using the chi-square test for linear trend. The height of $P_{10}$ was associated with treatment effectiveness using the Kruskal–Wallis test for non-normally distributed data.

### Results

A total of 52 patients with malignancy were studied. Primary tumour sites and histological types are shown in Table I. The most common primary sites were breast (22), colon (11) and lung (9). The most common histological type was adenocarcinoma (30). Patients ranged in age from 2 to 75 years (mean 55). Ten patients had received no treatment; for those who had received therapy, treatment was classified as ineffective for 10, partially effective for 17 and effective for 14 (Table I). A total of 94 patients were studied, which included healthy subjects, patients characterised as having non-malignant diseases and patients with various characterised infectious diseases. The patients with infectious diseases were also examined as controls.

*Identification of cancer-associated metabolites*

Four FPEC-GLC patterns were observed among the 52 specimens from cancer patients (Table I and Figures 1–4). These patterns were grouped as follows:

1. Group 1: Specimens contained a peak designated $P_1$ alone or in combination with a peak designated as $P_{10}$ shown in Figure 1a and b, Figure 2a and b and Figure 3a.
2. Group 2: Specimens contained $P_{10}$ along with additional peaks not found in controls, but found in advanced cancer cases (Figure 4a).
3. Group 3: Specimens contained $P_{10}$ and peaks associated with controls (Figure 4b).
4. Group 4: Specimens that had an FPEC-GLC profile that was like healthy controls without $P_1$ and $P_{10}$ (Figure 1c and Figure 2c).

$P_1$ was detected on an OV-1 column (Figure 1, chromatogram A). This peak was enhanced by extraction using a $C_{18}$ RPC column as seen by comparing computer-expanded portions of FPEC-GLC patterns (Figure 1a and b). $P_1$ was not found in the enhanced specimen from healthy controls (Figure 1c). It possessed unusual chromatographic characteristics in that the TCE ester did not elute on polar OV-225 or on the moderately polar OV-1701 capillary columns, and the ester slowly decomposed after standing for a month at 4°C. $P_1$ was extractable with chloroform under acidic but not basic conditions. It did not react with heptafluorobutyric anhydride either before or after esterification with TCE or PFB, and the TCE ester did not react with hydrogen in the presence of a platinum catalyst; therefore, $P_1$ was determined to be a saturated, short-chain, carboxylic acid without free amine or hydroxyl groups.

MS analysis of TCE-derivatised samples that contained large amounts (five times full scale) of $P_1$ by FPEC-GLC analysis was attempted on two occasions without obtaining meaningful spectra. We next used MS to analyse PFB-derivatised acidic chloroform extracts of sera taken from the same patients as the sera initially tested as TCE derivatives, and we were able to obtain mass spectra on $P_1$. The positive methane chemical ionisation mass spectrum of $P_1$ (Figure 5a) showed the protonated molecule at $m/z$ 271 and fragments at $m/z$ 243, 225 and 181 (base peak). The $m/z$ 181 fragment is also the base peak in the electron ionisation mass spectrum of $P_1$, which includes a subsequent hydrogen fluoride (HF) loss fragment at $m/z$ 161 but no molecular ion. The molecular weight is confirmed from its negative methane chemical ionisation mass spectrum whose base peak was the $m/z$ 89 fragment formed from dissociative electron attachment, resulting in the loss of the PFB derivative. Mass selection of the $m/z$ 89 anion with subsequent collisional activation and daughter ion analysis indicated two fragments at $m/z$ 43 and 45. From the spectra we concluded that $P_1$ was a monocarboxylic acid and that the underivatised acid had a molecular

**Figure 1** Frequency-pulsed electron-capture gas–liquid chromatography (FPEC-GLC) chromatograms of trichloroethanol (TCE) derivatives prepared from acidic cholorform extracts of serum taken from a patient with transitional cell carcinoma of the bladder. Chromatogram **a** shows peak 1 ($P_1$) before the sample was processed through the reversed-phase chromatography (RPC) column. Chromatogram **b** shows $P_1$ after RPC column clean-up, and chromatogram **c** is the control serum that, like **b**, also received RPC column clean-up. Only a segment of the chromatogram from 0 to 40 min is shown. The subscript number after the letter C indicates the number of carbon atoms, and the number after the colon is the number of unsaturated bonds. The letter i indicates a methyl group at the iso position in the carbon chain. Heptanoic acid ($C_7$) is the internal standard (IS) added to the serum sample prior to extraction and derivation.

**Figure 2** FPEC-GLC chromatograms of TCE derivatives prepared from acidic chloroform extracts of sera taken from two patients with early adenocarcinoma of the colon (chromatograms **a** and **b**; Table I, samples 7 and 8) and a suspected case of colon cancer that was later determined to be pancreatitis and had recovered to give an FPEC-GLC profile like a healthy control (chromatogram **c**). See legend to Figure 1 for definition of abbreviations. PAA, phenylacetic acid.

weight of 90. The following acids, which were considered as likely candidates for $P_1$, either did not co-elute with $P_1$ on the OV-1 column or failed to derivatise with TCE: methoxyacetic (the most likely candidate based on fragmentation studies), lactic, 3-hydroxypropionic and oxalic acids. Other compounds such as 1-ketobutyric, acetoacetic, ethyl-3-ethoxyacrylic and 2-methylbutyric acids succinicsemialdehyde were analysed by FPEC-GLC, but none of these co-eluted with $P_1$.

**Figure 3** FPEC-GLC chromatograms of TCE derivatives prepared from acidic chloroform extracts of sera. Chromatogram **a** was obtained by FPEC-GLC analysis of serum taken from a patient with early untreated squamous cell carcinoma of the nose, and chromatogram **b** was obtained by FPEC-GLC analysis of serum taken from the same patient shown in chromatogram **a** months after surgical removal of the cancer (Table I, samples 1 and 2). See legend to Figure 1 for definition of abbreviations.

**Figure 4** FPEC-GLC chromatograms of TCE derivatives prepared from acidic chloroform extracts of sera taken from a patient with adenocarcinoma of the sigmoid colon (chromatogram **a**). Therapy for 0.17 years (about 9 weeks) was ineffective and metastasis occurred. Chromatogram **b** (Table I, sample 37) was obtained by FPEC-GLC analysis of serum from a patient with progressive adenocarcinoma of the colon. See legend to Figure 1 for definition of abbreviations.

**Figure 5** Positive chemical ionisation gas–liquid chromatography mass spectra of $P_1$ (**a**) and $P_{10}$ (**b**). The PFB derivatives were prepared from acidic chloroform extracts of serum taken from a patient with advanced cancer.

We were also able to obtain electron impact and positive and negative chemical ionisation spectra on $P_{10}$. The positive methane chemical ionisation mass spectrum (Figure 5b) showed the protonated molecule at $m/z$ 601 with loss of pentafluorobenzylalcohol at $m/z$ 403 and the $m/z$ 181 fragment discussed previously. A weak fragment at $m/z$ 239 was evident, indicating the possible loss of two PFB molecules. The negative methane chemical ionisation spectrum showed the analogous $m/z$ 419 fragment, corresponding to the loss of the PFB derivative. Loss of carbon dioxide from the $m/z$ 419 ion yielded the $m/z$ 375 ion, and loss of a second PFB was noted at $m/z$ 239. Subsequent loss of carbon dioxide from the apparent second dicarboxylic fragment at $m/z$ 239 yielded the $m/z$ 195 fragment. The electron ionisation mass spectrum gave a strong molecular ion at $m/z$ 600 with the loss of PFB at $m/z$ 419, and the PFB fragment was the base peak at $m/z$ 181. A weak $m/z$ 238 was noted as well as the loss of carbon dioxide from $m/z$ 419 to give the $m/z$ 375 fragment. These data strongly indicated a molecular weight of 600 for the PFB derivative of peak 10.

**Figure 6** Electron impact spectra obtained from the mass spectral analysis of the trichloroethyl ester of $P_{10}$.

Similarly, analysis of the TCE derivative of $P_{10}$ using electron ionisation gave the bis-TCE derivative at $m/z$ 500. The isotopic distribution was that expected for the hexachloro derivative formed by attachment of two TCE reagents to $P_{10}$. Loss of trichloroethanol gave the $m/z$ 353 fragment with a significantly less complex isotopic distribution. Loss of 29 a.m.u. from $m/z$ 500 and $m/z$ 353 is apparently responsible for the $m/z$ 471 fragment and the $m/z$ 324 fragment. The negative methane chemical ionisation mass spectrum of $P_{10}$ confirmed both the electron impact spectrum of the bis-TCE derivative and that of the bis-PFB derivative data given in the preceding paragraph. The base peak of the negative chemical ionisation spectra at $m/z$ 369 showed an isotopic distribution containing three chlorines. A weak molecular ion, which contained the characteristic six-chlorine isotopic distribution, was seen at $m/z$ 500. The molecular weight of the bis-PFB derivative is 600, while that of the bis-TCE

**Figure 7** Proposed fragments obtained from mass spectral analysis of the trichloroethyl ester of $P_{10}$.

derivative is 500. Together, these data indicate a molecular weight of 240 for the underivatised $P_{10}$. The derivatised compound contains two TCE groups or two PFB groups, indicating that it is a dicarboxylic acid. The fragmentation pattern of the electron impact spectra (Figure 6) indicates a highly oxygenated compound with a very tentative structure presented (Figure 7). Evidence has been presented (McDonagh *et al.*, 1992) that monocytes and macrophages, in response to malignant tumours, release components that affect lipid metabolism and perioxidation was presented as one mechanism. $P_{10}$, like $P_1$, was not detected on the OV-225 polar column. In addition to peaks 1 and 10, several other peaks not found in controls were found in specimens from some cancer patients (Figure 4, peaks 3, 4, 7, 8 and 9). These substances were not always found in specimens that contain $P_{10}$ and they were usually seen in patients seriously ill with cancer. They have not been characterised further.

*Relationship of cancer-associated metabolites and clinical findings*

Of the specimens from 52 patients with malignancy, $P_1$ was identified in FPEC-GLC profiles of 17 (33%), $P_{10}$ in profiles of 37 (73%), and either peak in 42 (82%). When the entire profile was analysed, abnormalities were noted in 48 (94%) of the 52. Of the abnormal 92 control patients profiles, none contained $P_1$, $P_{10}$ or peaks associated with the cancer groups (Figure 4a). Thus, in our series, the sensitivity of FPEC-GLC for malignancy was 82% when considering only $P_1$ and $P_{10}$, or 94% when considering other abnormalities that occurred in the cancer groups, and the specificity was 100%. The quantity of $P_1$ or $P_{10}$ decreased with increasing effectiveness of therapy: sensitivity was 100% in profiles of patients who had received no therapy or ineffective therapy, 88% in those of patients whose therapy was partially effective and 50% in those of patients who had received effective therapy ($P<0.001$, chi-square for linear trend). No significant differences were seen in the proportion of samples containing these peaks by tumour site or type when the analysis was stratified for the effect of therapy.

Treatment had a significant effect on the presence of both $P_1$ and $P_{10}$. $P_1$ was present in profiles of 9 (90%) of 10 patients who had not received therapy compared with 8 (20%) of 41 patients who had received any therapy ($P<0.001$), regardless of its efficacy. In contrast, $P_{10}$ was present in most FPEC-GLC profiles of all treatment groups except for those with therapy classified as effective [34 (92%) of 37 patients with no treatment or ineffective or partially effective therapy versus 3 (21%) of 14 patients with effective therapy, $P = 0.001$]. The height of $P_{10}$ was also significantly less in the profiles of those who had received effective therapy compared with the other patient groups (4% full scale compared with 36% full scale, $P<0.001$). The effect of treatment on the FPEC-GLC profile was consistent regardless of tumour site and type.

### Discussion

The use of serum markers for the diagnosis and prognosis of cancer has been reviewed (Bates & Lono, 1987). Some of the markers extensively investigated for their value include carcinoembryonic antigen, alpha-fetoprotein, human chorionic gonadotrophic hormone and prostatic acid phosphatase. These markers most often are measured by radioimmunoassays (RIAs). While the assays detect nanogram levels of the markers, they lack the sensitivity and specificity to serve as screening tools for the early detection of cancer and have been most effective in disease management or for following the progression of previously diagnosed cancer.

More recently, the analysis of human plasma by nuclear magnetic resonance (NMR) was used to discriminate between healthy controls and cancer patients (Fossel *et al.*, 1986). However, further studies have indicated that this method lacks the specificity to serve as a test for the early diagnosis of cancer (Okunieff *et al.*, 1990; Shulman, 1990).

In the current study, detection of two peaks ($P_1$ and $P_{10}$) by FPEC-GLC of serum specimens was a sensitive and specific test for identifying patients with a variety of malignancies. Since FPEC-GLC can detect femtomole quantities of derivatised electron-absorbing compounds, it may be more sensitive than either RIA or NMR. Additionally, FPEC-GLC is specific for electron-absorbing compounds. Instrumentation for FPEC-GLC can be automated and is much less expensive than equipment needed for NMR analysis.

Results from selected chemical tests and mass spectroscopy suggest that $P_1$ is a monocarboxylic and $P_{10}$ a dicarboxylic acid and that both are highly oxygenated. While these results reflect progress in their characterisation, the true structural formula of these components remains to be developed. An accurate knowledge of their structure will be valuable in identifying the metabolic origin of these substances in patients with cancer.

Accurate mass measurements of the various ionic forms of $P_1$ and $P_{10}$ should be undertaken to establish their molecular formulae and to better define their fragmentation. Infrared spectroscopy in combination with gas chromatography may also provide some additional information regarding the nature of these compounds. Finally, MS in combination with liquid chromatography may be necessary to isolate sufficient quantitites of purified $P_1$ and $P_{10}$ for NMR analyses.

Peaks 1 and 10 cannot be absolutely quantitated on a molar basis by FLPEC-GLC without positive identification and comparison of the detector response to the actual compound because the response of the detector depends on the electron-absorbing characteristics of the derivatised compound; however, observations on peak concentration made from peak height and area obtained by comparison with an internal standard are valid. Although our study suggests that detection of specific peaks in specimens examined by FPEC-GLC was both sensitive and specific for patients with malignancy, the principal value of this procedure depends on its ability to detect malignancies early (before clinical signs or other diagnostic tests) or its ability to be used as a marker for the efficacy of therapy. Some data suggest that the presence and concentration of these metabolites vary with the success of therapy; however, the effect of therapy was not well quantified and serial specimens obtained during therapy from individual patients were not available. Prospective longitudinal studies of patients undergoing therapy are needed in order to validate this test's utility in monitoring the impact of treatment. Determining whether this procedure is useful as a diagnostic test might be accomplished by testing patients in groups at high risk for developing a malignancy and prospectively following patients with positive and negative test results for the development of cancer detected by standard methods.

FPEC-GLC tests on clinical samples of body fluids for tuberculous meningitis have been reported (Brooks *et al.*, 1990), and are presently being conducted by this laboratory. Further, the derivatisation technique used in the study has been refined and made practical, highly reproducible and reliable. Samples can be automatically injected into the gas chromatograph, and computers are available that expedite the handling of the data.

Results from this study indicate that FPEC-GLC may eventually offer the physician a means to screen and diagnose malignancies and to determine the effectiveness of cancer therapy.

The authors thank Dr C.W. Moss for his support of the project.

### References

BATES, S.E. & LONO, D.L. (1987). Use of serum markers in cancer diagnosis and management. *Semin. Oncol.*, **14**, 102–138.

BROOKS, J.B. (1986). Review of frequency-pulsed electron-capture gas-liquid chromatography studies of diarrheal disease caused by members of the family Enterobacteriaceae, *Clostridium difficile*, and rotavirus. *J. Clin Microbiol.*, **24**, 687–691.

BROOKS, J.B., CHERRY, W.B., THACKER, L. & ALLEY, C.C. (1972). Analysis by gas chromatography of amines and nitroamines produced *in vivo* and *in vitro* by *Proteus mirabilis*. *J. Infect. Dis.*, **126**, 143–153.

BROOKS, J.B., DANESHVAR, M.I., FAST, D.M. & GOOD, R.C. (1987). Selective procedures for detecting femtomole quantities of tuberculostearic acid in serum and cerebrospinal fluid by frequency-pulsed electron-capture gas–liquid chromatography. *J. Clin. Microbiol.*, **25**, 1201–1206.

BROOKS, J.B., DANESHVAR, M.I., HABERBERGER, R.I. & MIKHAIL, I.A. (1990). Rapid diagnosis of tuberculous meningitis by frequency-pulsed electron-capture gas-liquid chromatography of carboxylic acids in cerebrospinal fluid. *J. Clin. Microbiol.*, **28**, 989–997.

DANESHVAR, M.I. & BROOKS, J.B. (1988). Improved procedure for preparation of pentafluorobenzyl derivatives of carboxylic acids for analysis by gas chromatography with electron capture detection. *J. Chromatogr.*, **433**, 248–256.

ENGAN, T., KRANE, J., KLEPP, O. & KNINNSLAND, S. (1990). Proton nuclear magnetic resonance spectroscopy of plasma from healthy subjects and patients with cancer. *N. Engl. J. Med.*, **322**, 949–953.

FOSSEL, E.T., CARR, J.M. & MCDONAGH, J. (1986). Detection of malignant tumors by water-suppressed proton nuclear magnetic resonance spectroscopy of plasma. *N. Engl. J. Med.*, **315**, 1369–1376.

GARRETT, P.E. & KURTGIS, R. (1985). Clinical utility of oncofetal proteins and hormones as tumor markers. Update on diagnostic technique. *Med. Clin. N. Am.*, **70**, 1295–1306.

MCDONAGH, J., FOSSEL, E.T., KRADIN, R.L., DUBINETT, S.M., LAPOSATA, M. & HALLAF, Y.A. (1992). Effects of tumor necrosis factor-α on perioxidation of plasma lipoprotein lipids in experimental animals and patients. *Blood*, **80**, 3217–3226.

OKUNIEFF, P., ZEITMAN, A., KHAN, J., SINGER, S., NEWRINGER, L.J., LEUINE, R.A. & EMANS, F.E. (1990). Lack of efficacy of water-suppressed proton nuclear magnetic resonance spectroscopy of plasma for the detection of malignant tumors. *N. Engl. J. Med.*, **322**, 953–958.

SELL, S. (1990). Cancer markers of the 1990s. Comparison of the new generation of markers defined by monoclonal antibodies and oncogene probes to prototype markers. *Clin. Lab. Med.*, **10**, 1–37.

SHULMAN, R.S. (1990). NMR another cancer test disappointment. *N. Engl. J. Med.*, **322**, 1002–1003.

SONDIK, E. (1988). *NCI Annual Cancer Statistics Review*. NCI internal publication. NCI: Bethesda, MD.